# Epidemiology and Outcomes of Neonatal Hemophagocytic Lymphohistiocytosis

**DOI:** 10.3389/fped.2022.848004

**Published:** 2022-04-26

**Authors:** Niveditha Balakumar, Prithvi Sendi, Balagangadhar R. Totapally

**Affiliations:** ^1^Division of Critical Care Medicine, Children's Hospital of San Antonio, Baylor College of Medicine, San Antonio, TX, United States; ^2^Division of Critical Care Medicine, Nicklaus Children's Hospital, Miami, FL, United States; ^3^Herbert Wertheim College of Medicine, Florida International University, Miami, FL, United States

**Keywords:** HLH, hemophagocytic lymphohistiocytosis, neonatal, sepsis, multi organ dysfunction

## Abstract

**Objectives:**

Neonatal hemophagocytic lymphohistiocytosis (HLH) is a rare entity. The objective of the study was to describe the prevalence, clinical characteristics, interventions and outcomes of neonates diagnosed with HLH in the United States.

**Methods:**

A retrospective analysis of 2009, 2012, and 2016 Kids' Inpatient Database was performed. Neonates discharged/died with a diagnosis of HLH were identified and analyzed.

**Results:**

Among 11,130,055 discharges, 76 neonates had a diagnosis of HLH. Fifty-two percent (95% CI: 38.6–63.6) were males and 54% (95% CI: 39.7–68.5) were white. Herpes simplex infection was present in 16% (95% CI: 9.2–28.1). 24.4% (95% CI: 14.5–37.9) received chemotherapy, 11.5% (95% CI: 5.2–23.6) IVIG and 3.6% (95% CI: 0.8–14.4) allogenic hemopoietic stem cell transplantation. Organ dysfunction was commonly seen and severe sepsis was documented in 26.6% (95% CI: 16.4–39.9). Median LOS was 16 (IQR 7–54) days. The mortality was 42% (95% CI: 30.8–55).

**Conclusions:**

HLH is a rare diagnosis and carries a high mortality in neonates. Herpes simplex virus is the most common infection associated with neonatal HLH. HLH should be considered in the differential diagnosis in neonates presenting with multi-organ dysfunction or sepsis.

## Introduction

Hemophagocytic lymphohistiocytosis (HLH) is a rare life-threatening, severe inflammatory disorder in children. In HLH, activation and extreme dysregulation of macrophages and T cells occur resulting in pathological inflammation. HLH can occur as a genetic condition (primary/familial) or secondary to infection, malignancy or rheumatologic conditions. The familial types are usually diagnosed in infancy or childhood, while secondary HLH can occur at any age (1).

As a part of the HLH-94 clinical trial, the Histiocyte Society proposed a standard definition of HLH in 1994 (2). The definition was later revised for the HLH-2004 trial and serves as a current definition of HLH (3). In the first prospective international treatment study published in 1994, five criteria for HLH diagnosis were included, namely, fever, splenomegaly, bicytopenia, hypertriglyceridemia and/or hypofibrinogenemia, and hemophagocytosis. The treatment combined chemotherapy and immunotherapy (etoposide, corticosteroids, cyclosporin A, and, in selected patients, intrathecal methotrexate), followed by bone marrow transplantation (BMT) in persistent, recurring, and/or familial disease (2). In 2004, three additional criteria were introduced including low/absent NK-cell-activity, hyperferritinemia, and high-soluble interleukin-2-receptor levels. Five of the eight criteria must be fulfilled, unless family history or molecular diagnosis is consistent with HLH (3). In 2019 North American Consortium for Histiocytosis proposed HLH disease should be distinguished from HLH disease mimics. They suggest HLH subtypes should be categorized by specific etiologic association and not by usual classification of primary and secondary HLH (4).

Hemophagocytic lymphohistiocytosis occurring within first 4 weeks after birth is a rare entity. The incidence of neonatal HLH is not known and it may range from 1 in 50,000 to 150,000 (5). The incidence varies based on ethnicity, especially in populations in which consanguinity is common (5, 6). A high index of suspicion is necessary to diagnose neonatal HLH as its presentation is similar to any other sick neonates. It is often missed, with the diagnosis being made only after an autopsy (7). Most of the reports of neonatal HLH are either case reports or small case series which have shown a wide range of presentation including hydrops fetalis, respiratory distress, thrombocytopenia, sepsis or multi organ failure (8–11). The objective of this study is to describe the prevalence, clinical characteristics, interventions and outcomes of neonates diagnosed with HLH in the United States.

## Methods

A retrospective review of the Healthcare Cost and Utilization Project (HCUP) Kid's Inpatient Database (KID) for the years 2009, 2012, and 2016 was performed. The KID is a nationwide database that falls under the umbrella of HCUP databases which are sponsored by the Agency for Healthcare Research and Quality (AHRQ). The KID can be used to identify, track and analyze national trends in health care utilization, charges, quality, and outcomes. It excludes data elements that could directly or indirectly identify individuals. The included data has been provided by state agencies across the nation every 3 years since inception. The KID includes 4,121, 4,179, and 4,200 hospitals for the years 2009, 2012, and 2016, respectively. Forty-four states contributed to the database in 2009 and 2012, and 46 states in 2016.

The merged databases from 2009, 2012, and 2016 yielded a total of 11,130,055 neonatal discharges. The International Classification of Diseases, Ninth and Tenth Revision, Clinical Modification (ICD-9 and ICD-10) diagnosis and procedure codes were used for HLH and other variables. The Database was queried for neonatal discharges with a diagnosis of HLH. We used ICD-9 code, 288.4, during 2009 and 2012 and ICD-10 codes, D76.1, D76.2, and D76.3, during 2016 for identification of HLH. Demographic and clinical characteristics, interventions and outcome variables were extracted for further analysis. Total charges in 2009 and 2012 were adjusted for inflation to 2016 values^1^.

## Statistical Analysis

Complex sampling and data weighting (for national estimates) were employed for all analyses. Length of stay and total charges, are described as medians with interquartile range (IQR). Univariate and multivariable analyses were used to analyze mortality outcome. Variables with a *p*-value of <0.1 by univariate analysis were included in the binary regression model. IBM SPSS Statistics for Windows, version 25 (IBM Corp., Armonk, N.Y., USA) was used for data analysis, and a *p*-value < 0.05 was considered significant. The study was considered exempt by the Institutional Review Board.

## Results

### Prevalence and Demographic Characteristics

Among a total of 11,130,055 neonatal discharges, 76 neonates were discharged with a diagnosis of HLH. The prevalence of neonatal HLH was 0.06 per 10,000 discharges. Males comprised of 52% (95% CI: 38.6–63.6) and 54% (95% CI: 39.7–68.5) were whites, 21.1% (95% CI: 12.3–33.8) were black, and 8% (95% CI: 3.4–20.9) were Hispanics (Table 1).

**Table 1:** Demographic characteristics in neonates with hemophagocytic lymphohistiocytosis.

	**% HLH discharges (95% CI)**
**Gender**	
Male	51.2 (38.6–63.6)
**Race**	
White	54.5 (39.7–68.5)
Black	21.1 (12.3–33.8)
Hispanic	8.8 (3.4–20.9)
Asian/Pacific islander	4.5 (1.1–17.4)
Other	11 (4.7–23.9)

### Clinical Characteristics

**Figure 1:**
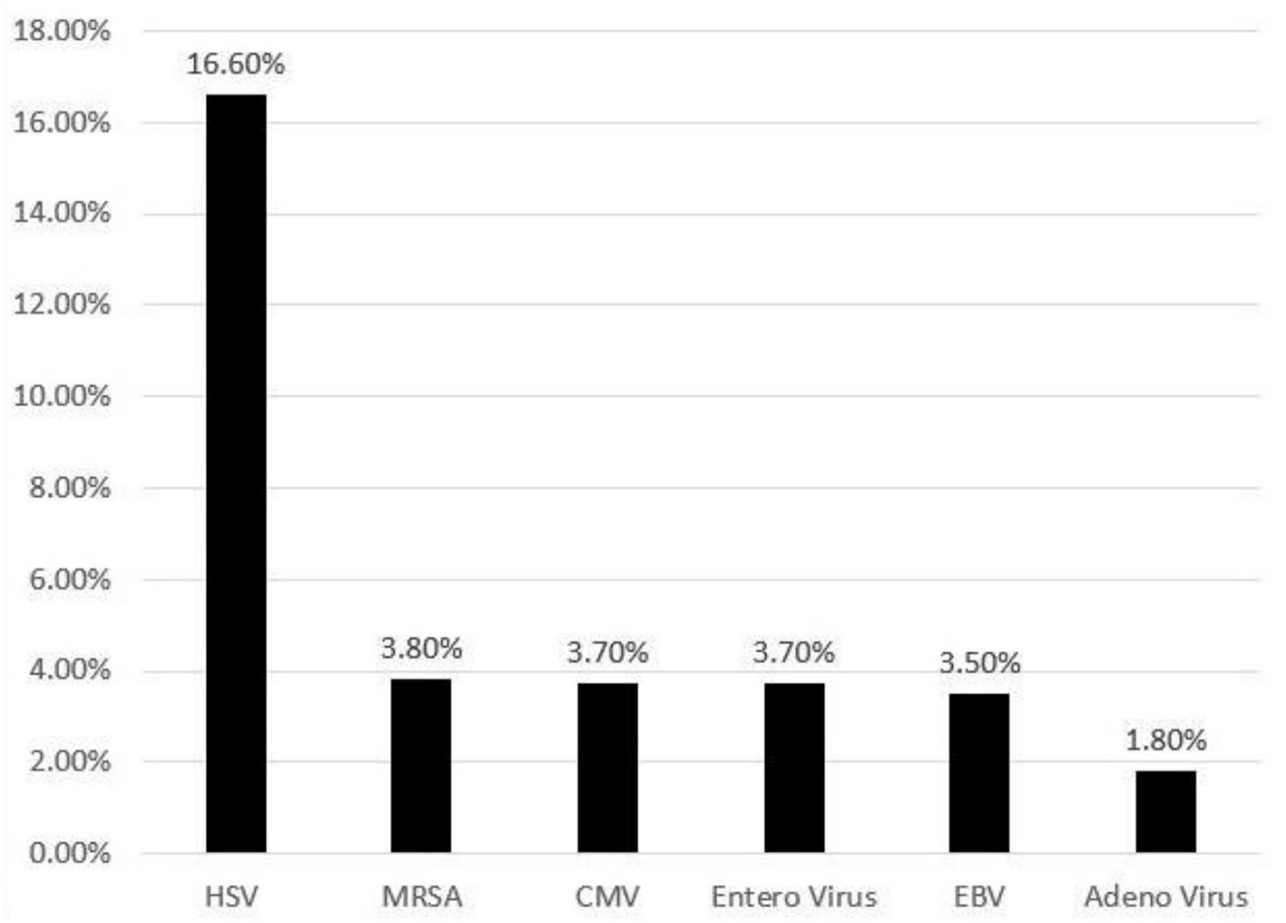
Frequency (%) of various infections identified in neonates with hemophagocytic lymphohistiocytosis.

Herpes simplex virus (HSV) infection was documented in 16% (95% CI: 9.2–28.1) of HLH discharges followed by other infections such as, cytomegalovirus (CMV), enterovirus, Ebstein-Barr virus (EBV), adenovirus and bacterial infections like methicillin resistant *Staphylococcus aureus* (Figure 1). Immunodeficiency was seen in 3.5% of neonates with HLH. The prevalence of various organ dysfunction is presented in Table 2.

**Table 2:** Frequency of organ dysfunction and interventions and associated mortality in neonates with hemophagocytic lymphohistiocytosis.

**Organ dysfunction**	**% of HLH discharges (95% CI)**	**% Mortality (95% CI)**	**OR (95% CI)**	***p-*** **value**
Hydrops fetalis	20.8 (12–33.8)	63.6 (33.5–95)	2.99 (0.74–12.06)	0.11
Bone marrow dysfunction	16.4 (8.5–29.3)	33.0 (10.6–67.1)	0.61 (0.13–2.88)	0.52
Liver dysfunction	35 (23.8–49.4)	67.5 (44.1–84.6)	5.18 (1.42–18.8)	0.01
Renal Failure	55.3 (44.0–65.9)	39.7 (25.2–56.3)	0.77 (0.27–2.23)	0.63
Severe sepsis	26.6 (16.4–39.9)	64.8 (36.7–85.3)	3.51 (0.92–13.29)	0.05
**Interventions**			
ECMO	3.7 (0.9–14.6)	47.3 (4.9–94)	1.28 (0.06–21.96)	0.88
Mechanical ventilation	67 (54.0–78.0)	63.3 (46.1–77.7)	2.68 (1.88–3.83)	0.00
IVIG	11.5 (5.2–23.6)	65 (22.7–96)	2.93 (0.40–21.34)	0.26
Anti-neoplastic agent	24.4 (14.5–37.9)	36.1 (16.7–61.5)	0.70 (0.22–2.26)	0.55
HSCT	3.6 (0.8–14.4)			

*ECMO, Extracorporeal membrane oxygenation; IVIG, Intravenous immunoglobulin; HLH, hemophagocytic lymphohistiocytosis; HSCT, Hematopoietic stem cell transplant*.

### Diagnostic and Therapeutic Interventions

Bone marrow biopsy was documented in 33% (95% CI: 21.2–47.7) of neonates with HLH. Dialysis was done in 9.3% (95% CI: 4.3–18.7) and extracorporeal membrane oxygenation support was provided in 3.7% (95% CI: 0.9–14.6). Chemotherapy was administered in 25% (95% CI: 14.5–37.9) and IVIG in 11.5% (95% CI: 5.2–23.6). Allogeneic hemopoietic stem cell transplantation was done in 3.6% (95% CI: 0.8–14.4; Table 2).

### Outcomes

The overall mortality for the cohort was 42% (95% CI: 30.8–55.0). A total of 41% (95% CI: 30.2–53.2) of neonates with HLH were discharged home and 12% (95% CI: 6.1–24.7) required home health care. On univariate analysis, factors associated with increased mortality are presented in Table 2. On multivariable analysis only the presence of liver dysfunction was associated with increased mortality [OR: 6.5 (95%CI: 1.1–37.7)]. The median length of stay for the entire cohort was 16 (IQR: 7–54) days. The median hospital charges were $ 258,757 (IQR: 126,860–638,578).

## Discussion

Neonatal HLH is a rare condition with a prevalence of 0.06 per 10,000 neonatal discharges in our national cohort. Most reports of neonates with HLH have been a part of childhood HLH studies (2), or case series (8–12). To the best of our knowledge we are reporting the largest cohort of neonatal HLH.

Clinical and laboratory characteristics for neonatal HLH are not well studied. Several infections were noted to be associated with HLH in our study as well as previous publications. In a previously published study evaluating 20 neonates with the diagnosis of HLH, herpes simplex virus (HSV) was the most commonly seen infection similar to our study (8). In another report of neonatal HLH, 7 out of 9 neonates had infection-triggered HLH with either HSV or entero virus (9). In addition, Takehara et al. also reported a case of a neonate with disseminated HSV-1 infection complicated by HLH and multi-organ failure which was ultimately fatal (10). There are case reports of adenovirus triggered HLH in two neonates who eventually developed acute respiratory distress syndrome and cardiovascular failure before succumbing to illness despite being on extracorporeal membrane oxygenation support (11). Secondary HLH triggered by other infections like CMV (12) and entero virus (13) have also been reported.

Our study highlighted the high occurrence of multi-organ dysfunction in neonates with HLH. A multitude of presentations have been described including fever, respiratory distress, thrombocytopenia, opisthotonos and sepsis (8, 9, 11, 14). Acute liver failure could be the first presenting sign of HLH (15). In our study, the presence of liver dysfunction was associated with increased mortality in multivariable analysis. We were unable to determine any specific criteria used to diagnose HLH like ferritin levels, soluble IL-2 receptor and NK cell activity due to lack of specific ICD codes in the database.

Several case reports on familial neonatal HLH have found hydrops fetalis and multi organ failure as a common presentation. In cases of familial HLH, symptoms are evident within first few months after birth, or even *in utero* (7, 14). Malloy et al. reported a case of intrauterine HLH in an infant who presented with hydrops fetalis and pancytopenia at 32 weeks gestation which was ultimately fatal (16). In 2009, Vermeulen et al. published a case report of twins with familial HLH caused by a PRF1 mutation where one baby had hydrops fetalis *in utero* and the second presented soon after birth with fatal multiple organ failure (17). Gurgey et al. reported primary HLH in 8 neonates in Turkey (18). Mutational analyses were performed in 7 of these patients. UNC13D gene mutations were detected in 3 of the patients, two of the patients were found to have perforin gene mutation. All these patients were symptomatic in the first 10 days of life. Familial HLH should be included in the differential diagnosis of non-immune hydrops fetalis and neonatal multiple organ failure. Our study involving administrative database does not contain ICD codes for diagnosis for familial HLH; however, given the occurrence of hydrops fetalis in 20% of the neonates in our study, it can be deduced they likely had familial HLH.

Determining the cause of HLH whether primary or secondary is important for early treatment. Chemo-immunotherapy for HLH includes etoposide, dexamethasone and cyclosporine A. Hematopoietic stem cell transplantation (HSCT) is recommended for patients with familial disease and patients with severe and persistent, or reactivated disease (3). If the cause is determined to be familial, chemo-immunotherapy can provide temporary control of the disease, however HSCT is the treatment of choice since it is curative (2, 3). Only 3.6% neonates received HSCT in our series. Neonates who were discharged and subsequently received HSCT could not be identified in our study.

If the cause is deemed to be secondary HLH, identifying the cause and starting early chemo-immunotherapy is of utmost importance. Considering that HSV is the most common infectious agent, prompt diagnosis and treatment with high dose acyclovir is essential. We could not identify any patient who received steroid or individual specific medication due to database limitations. Although rare, neonatal HLH carries a high mortality as evidenced by a mortality rate of 42% in our study. Suzuki et al. also showed a similarly high mortality of 60% in their case series (8).

Imaging such as PET/CT has been shown to be useful in identifying triggers for Secondary HLH like infection or malignant disease and also evaluate extent of secondary HLH (19). In several case reports PET/CT was useful not only for staging in familial HLH but also able to evaluate the therapy response (20, 21).

## Limitations

Large discharge databases, such as the KID, are useful for studying rare conditions like neonatal HLH. However, there are several limitations of studies using administrative databases. Validity of the studies using administrative databases can be questioned due to coding errors like, omissions or inaccurate coding. In addition, chronological relationships are often difficult to assess. Moreover, if some risk factors that may be present but cannot be identified, may lead such studies to be misinterpreted. In our study due to lack of ICD codes for familial HLH, we couldn't differentiate familial vs. secondary HLH. We were unable to determine which of the HLH-2004 diagnostic criteria were applied for diagnosis of HLH in our study. Individual medications that were used for the management of HLH could not be identified. We were not able to differentiate whether associated infections are primary precipitating cause of HLH or secondary infections occurring during the management of HLH. Due to database limitations we could not identify any imaging studies done for our patients. Additionally, follow-up information for neonates who were discharged from the hospital is not available. Despite the limitations of the study, our data sheds light on the demographic details and outcomes of neonates with HLH.

## Conclusions

Hemophagocytic lymphohistiocytosis is a rare condition in neonates and carries a high mortality. Herpes simplex virus is the most common infection associated with neonatal HLH. The presence of liver dysfunction is associated with increased mortality. HLH should be included in the differential diagnosis of neonates presenting with multi organ dysfunction or sepsis. Increased awareness of this condition among neonatologists is important for early diagnosis and prompt hemato-oncology consultation and treatment.

## Data Availability Statement

The original contributions presented in the study are included in the article/supplementary material, further inquiries can be directed to the corresponding author.

## Author Contributions

NB: data collection and literature review. PS: data collection and statistics. BT: data collection, statistics, and editing. All authors contributed to the article and approved the submitted version.

## Conflict of Interest

The authors declare that the research was conducted in the absence of any commercial or financial relationships that could be construed as a potential conflict of interest.

## Publisher's Note

All claims expressed in this article are solely those of the authors and do not necessarily represent those of their affiliated organizations, or those of the publisher, the editors and the reviewers. Any product that may be evaluated in this article, or claim that may be made by its manufacturer, is not guaranteed or endorsed by the publisher.

## Footnotes

^1^ www.bls.gov/cpi/tables/supplementalfiles/historicalcpi-u-202002.pdf
